# Assessment of Preventive Practices Followed by General Public During COVID-19 Pandemic - A Cross-Sectional Survey From India

**DOI:** 10.7759/cureus.11274

**Published:** 2020-10-31

**Authors:** Avinash Chakrawarty, Piyush Ranjan, Arnav Thrinath, Eishvauk Aggarwal, Joshua A Isaac, Parul Berry, Upendra Baitha, Ashish D Upadhyay, Souradeep Chowdhury, Arvind Kumar

**Affiliations:** 1 Geriatric Medicine, All India Institute of Medical Sciences, New Delhi, IND; 2 Medicine, All India Institute of Medical Sciences, New Delhi, IND; 3 Statistics, All India Institute of Medical Sciences, New Delhi, IND

**Keywords:** covid 19, cross-sectional survey, preventive practices, general population, india

## Abstract

Objectives

COVID-19 has infected millions of people across the globe, leading to hundreds of thousands of deaths. Currently, there are no vaccines available for COVID-19, and the most effective way to curb its spread is to follow preventive practices. The present study aimed to assess the extent of adoption of preventive practices among the general population in India.

Methods

A web-based cross-sectional survey was carried out recruiting 964 participants from all over India through purposive sampling. A pre-validated questionnaire consisting of 37 questions was used to collect data. Items 1A to 18A covered various preventive practices and items 1B to 19B covered reasons for not following those preventive practices. Descriptive statistics, chi-square tests, t-tests and one-way analysis of variance (ANOVA) were conducted.

Results

Most participants reported taking precautions such as wearing masks (91.80%), covering both nose and mouth (79.14%) and avoiding hand shaking (83.40%). However, practices like following social distancing in public places (51.76%) and workplace (51.04%), frequent hand washing/sanitising (63.59%) and washing hands for at least 20 seconds (45.44%) were less commonly observed. Participants failed to follow social distancing because of overcrowding and lack of space. They also found it cumbersome to wash hands multiple times. Female participants and people residing in metropolitan and small cities were fairly doing well in following preventive practices.

Conclusion

The study helped in identifying the glitches in following various preventive practices against COVID-19 during unlock phase and reasons for the failure to perform these practices.

## Introduction

COVID-19 has led to an unprecedented public health crisis across the globe. Millions of people worldwide have been infected with the virus with hundreds of thousands of people dying of it [1]. In India too, the number of cases has reached millions with the number of deaths surging day by day. Many of these cases are active and pose a risk of transmission to other citizens [2]. Currently, there are no specific treatments or vaccines for COVID-19. Moreover, the use of hydroxychloroquine for pre-exposure prophylaxis in COVID-19 is also debatable [3]. Thus, the most effective way to curb the transmission of this disease is to religiously adopt and follow various preventive practices against COVID-19 [4-6].

Public health authorities are continually emphasising the need to adopt certain preventive practices related to hand and personal hygiene, social distancing, avoiding unnecessary travel, etc. Several factors like diverse cultures, wide socioeconomic disparities and healthcare services inequalities might pose challenges in implementation of these preventive practices at the community level [7]. This calls for a need to assess the awareness among people as well as actual practice being followed regarding preventive practices against COVID-19.

There is a paucity of studies conducted in India to assess preventive practices being followed by people against COVID. The objective of our study was to assess the level of awareness and adoption of various preventive practices against COVID-19 among people of different age groups, socioeconomic status and residing in different regions of the country.

## Materials and methods

Study design and setting

We conducted this web-based cross-sectional study between 30th July 2020 and 30th August 2020 covering different states of India. The study was approved by the institute’s ethics committee. Relying on the investigators’ contacts with local people, purposive and snowball sampling techniques were used to obtain study participants from all over India. Informed consent was obtained from all participants at the time of enrolment in the study.

Study participants and data collection

Participants aged 18 years and above, residing in different parts of India and belonging to different socio-economic groups were recruited in the study. Data was collected using online platforms and telephonic interviews. The weblink of the questionnaire was sent through email and WhatsApp messenger. Our Google form consisted of a ‘Participant Information Sheet’ containing brief information about the objectives, expected duration of participation, potential risks/benefits, voluntary nature of participation and declaration of confidentiality. Participants providing informed consent by clicking on the checkbox were directed to the main questionnaire. Confidentiality and anonymity of all participants were maintained. Telephonic interviews were carried out by the investigators for those who could not fill the questionnaire online, and the data was entered into the excel sheet simultaneously.

Research tool

A 37-item questionnaire developed and validated in the previous research [8] by following the standardized methodology [9] was used for data collection. Socio-demographic details including age, gender, education, occupation, the city currently residing in, marital status, education and occupation of the head of the family, monthly income of the family were obtained. The questionnaire consisted of two sections: section A comprising items 1A to 18A covering preventive practices under various domains such as hand hygiene, social distancing, use of masks, gadgets, technology, etc. A 5-point Likert scale was used with the response options as ‘Always (more than 90% times)’, ‘Mostly (approx. 75% times)’, ‘Commonly (approx. 50% times)’, ‘Occasionally (approx. 25% times)’ and ‘Rarely (less than 10% times)’. For all items except 1A,10A,17A,18A, ‘Always (more than 90% times)’ was considered as the ideal response, ‘Mostly (approx. 75% times)’ as acceptable and rest of the response options were considered as unacceptable whereas for item 1A reverse scoring was done. For item 10A, the response option ‘Never’ was an ideal response, ‘Once’ was acceptable whereas other response options were considered to be unacceptable. For items, 17A and 18A the response options were ‘Strongly agree’, ‘Agree’, ‘Can’t say’, ‘Disagree’ and ‘Strongly disagree’ wherein ‘Strongly agree’ was considered as an ideal response, ‘Agree’ as acceptable and other response options as unacceptable. Section B of the questionnaire consisted of items 1B to 19B including questions regarding reasons for deficiency in following preventive practices. The face validity and content validity of the questionnaire were assessed both qualitatively and quantitatively. The Cronbach’s alpha coefficient of the questionnaire was 0.82 suggesting its good internal consistency and reliability.

Statistical analysis

Responses received through the Google form were obtained as excel sheet. Data was coded and then analysed using IBM® SPSS® Statistics version 24.0 software (IBM Corp., Armonk, NY). Each qualitative variable was expressed as an absolute and relative frequency, whereas the continuous variables were expressed as mean along with standard deviation. The association between qualitative variables was found using the chi-square test or Fisher exact test according to the distribution of data. Continuous variables were compared by independent t-test (two groups) and one way ANOVA followed by post-hoc comparison using Bonferroni test/Kruskal-Wallis test followed by multiple comparison using Dunn test with Bonferroni correction (more than two groups). A p-value of less than 0.05 was considered to be statistically significant.

## Results

Demographic characteristics

A total of 984 participants submitted their responses, of which responses from 20 participants were removed due to incomplete information. The final sample comprised 964 participants with a fair representation of people from different age groups, gender, socio-economic groups, educational background and parts of the country. The socio-demographic details are included in Table 1.

**Table 1:** Socio-demographic profile (n = 964)

Demographic characteristics	Respondents [Frequency (%)]
Age (years) (Mean 40 ± 14.8 SD)	
18-30	307 (31.85)
31-50	424 (43.98)
51 and above	233 (24.17)
Gender	
Male	555 (57.57)
Female	409 (42.43)
Marital status	
Single	293 (30.39)
Married	666 (69.09)
Others (divorced, etc.)	5 (0.52)
Socioeconomic status	
Upper	310 (32.16)
Middle	339 (35.17)
Lower	315 (32.68)
Place of residence	
Metropolitan cities	516 (53.53)
Small cities	313 (32.47)
Small towns and villages	135 (14.00)

Assessment of adoption of preventive practices

All the domains of preventive practices such as hand hygiene, social distancing, use of masks and attending social gatherings were assessed by the 18 questions of the validated questionnaire. The frequency of responses given by the participants to various questions has been presented in Table 2.

**Table 2:** Frequency of responses to various items in section A of the questionnaire (n = 964) Except items 1, 10, 17, 18, other items are rated as (1) = Rarely (less than 10% times), (2) = Occasionally (approx. 25% times), (3) = Commonly (approx. 50% times), (4) = Mostly (approx. 75% times), (5) = Always (more than 90% times). For item 1: (1) = Always (more than 90% times), (2) = Mostly (approx. 75% times), (3) = Commonly (approx. 50% times), (4) = Occasionally (approx. 25% times), (5) = Rarely (less than 10% times). For item 10: (1) = More than three times, (2) = Thrice, (3) = Twice, (4) = Once, (5) = Never.

S No.	Item	Frequency of responses (%)				
		1	2	3	4	5
Hand hygiene						
	How often do you shake hands while greeting people now-a-days?	51 (5.29)	24 (2.49)	26 (2.70)	59 (6.12)	804 (83.40)
	How often do you wash/sanitize your hands with soap and water/alcohol based sanitizer?	5 (0.52)	17 (1.76)	60 (6.22)	269 (27.90)	613 (63.59)
	How often do you ensure that you wash/sanitize your hands for at least 20 seconds?	25 (2.59)	41 (4.25)	138 (14.32)	322 (33.40)	438 (45.44)
	How often do you ensure that you clean your hands before touching your eyes/nose/mouth?	35 (3.63)	65 (6.74)	159 (16.49)	359 (37.24)	346 (35.89)
	How often do you ensure that you cover your face with a handkerchief/bent elbow while coughing/sneezing?	17 (1.76)	17 (1.76)	66 (6.85)	208 (21.58)	656 (68.04)
Social distancing						
	How often do you maintain a minimum distance of 1 metre at your workplace?	17 (1.76)	24 (2.49)	115 (11.93)	316 (32.78)	492 (51.04)
	How often do you maintain a minimum distance of 1 metre while eating food with your colleagues at your workplace?	41 (4.25)	47 (4.88)	106 (11.00)	263 (27.28)	507 (52.59)
	How often do you avoid going out of the house unnecessarily?	51 (5.29)	32 (3.32)	63 (6.54)	261 (27.07)	557 (57.78)
	How often do you maintain a minimum distance of 1 metre in public spaces (Ex: grocery shopping, social gatherings, etc.)?	11 (1.14)	23 (2.39)	117 (12.14)	314 (32.57)	499 (51.76)
	How often have you attended social gatherings in the past two months? (Like meeting friends, going to religious places, visiting malls, theatres, etc.)?	62 (6.43)	55 (5.71)	103 (10.68)	166 (17.22)	578 (59.96)
Masks						
	How often do you wear mask while going out of home?	1 (0.10)	4 (0.41)	12 (1.24)	62 (6.43)	885 (91.80)
	While wearing a mask, how often do you ensure that both your nose and mouth are covered?	2 (0.21)	4 (0.41)	42 (4.36)	153 (15.87)	763 (79.14)
	How often do you keep your mask properly in a separate bag/dustbin after using it?	46 (4.77)	46 (4.77)	83 (8.61)	174 (18.05)	615 (63.80)
Gadgets/Fomites						
	How often do you clean/sanitise your personal items (e.g., purse/mobile phone, etc.) with sanitizer when you come home?	118 (12.24)	93 (9.65)	151 (15.66)	252 (26.14)	350 (36.31)
	How often do you take precautions when buying things to avoid virus contamination?	13 (1.35)	30 (3.11)	102 (10.58)	277 (28.73)	542 (56.22)
Law						
	How often do you obey government restrictions regarding COVID pandemic?	4 (0.41)	7 (0.73)	44 (4.56)	217 (22.51)	692 (71.78)
	In case you develop symptoms of disease, will you contact hospital/helpline/authority regarding it?	1 (0.10)	9 (0.93)	54 (5.60)	173 (17.95)	727 (75.41)
	If you come in contact with COVID positive/suspect person, would you stop going to work and confine yourself to a home away from friends and family members?	4 (0.41)	7 (0.73)	32 (3.32)	143 (14.83)	778 (80.70)

Three-fifths of the participants reported that they avoided going out of the house and have not attended any social gathering in the last two months. More than 90% (885) of the participants reported that they wore masks most of the time when outside home but only 80% (763) of them ensured wearing masks properly. With respect to social distancing, nearly half of the participants reported that they strictly followed social distancing at public places, workplace and while having food with their colleagues, etc.

When asked about the hand hygiene, around two-third participants reported the regular use of soap and water/alcohol-based sanitizer to clean their hands. However, less than half ensured hand washing for at least 20 seconds. After coming home, nearly two-third participants ensured keeping their masks properly in a separate bag/dustbin after using it but only about one-third of the participants reported that they clean/sanitize their personal items (e.g., purse/mobile phone, etc.) with sanitizer after coming home. More than 80% participants (804) reported avoiding hand shaking.

The number of participants giving ideal responses to various questions is depicted in Table 3. Majority (80%) of participants gave an ideal answer that they wore masks and avoided shaking hands. 60-80% were practising proper hand hygiene and ensured covering both the nose and mouth with the mask. On the other hand, only 40-60% of participants confirmed that they washed/sanitized their hands for at least 20 seconds and followed social distancing. Moreover, less than 40% of the participants ensured cleaning hands before touching eyes/nose/mouth and cleaning/sanitizing personal items (e.g., purse/mobile phone, etc.) with sanitizer after returning home.

**Table 3:** Percentage of participants giving ideal response for various items

Frequency of responses to items	No. of items	Questions
>80% participants giving ideal response	3	1,11,18
60-80% participants giving ideal responses	6	2,5,12,13,16,17
40-60% participants giving ideal responses	7	3,6,7,8,9,10,15
<40% participants giving ideal responses	2	4,14

Assessment of reasons for deficiency in following preventive practices

Participants were enquired about various reasons for missing out on stringently following certain preventive practices presented in the appendix (Table 5).

Hand hygiene

Participants at times could not resist shaking hands as not doing so appeared rude to them. They also found it difficult to change the long-standing habit. They missed out washing hands at frequent intervals for at least 20 seconds as they were unable to always check the time and found it cumbersome.

Social distancing

Participants whenever they go out for grocery shopping, work and walking/exercising, they sometimes failed to maintain social distance either because of overcrowding or lack of space.

Masks

Participants sometimes missed out wearing masks and covering their nose and mouth when going out of the house due to difficulty in breathing and also it slid down due to its loose fit. Moreover, there were participants who had no clue where and how to dispose of the used masks.

Gadgets/fomites

Participants felt that using sanitizer on personal items like mobile will damage it or they thought it is not needed and hence, refrained from doing so. On the other hand, participants took various precautions while purchasing groceries from local stores/vendors such as wearing a face mask, buying 1-2 weeks-worth of groceries at a time, carrying hand sanitizer or wipes along with them, shopping at a time when it is less busy, using mobile pay/debit cards/credit cards for making payments or opting for home delivery.

Technology and law

More than half of the participants had installed AarogyaSetu app in their phones, however, they did not use it as they did not find it to be very useful.

Preparedness

More than 80% of participants were aware of any helpline/authority/hospital to contact in case they develop symptoms of disease. However, many preferred to treat themselves at home if they develop symptoms.

Causes and solutions for COVID-19 spread - Participants’ perception

Participants were also asked about their views regarding the cause of the rising number of COVID cases among the general population and their solutions presented in detail in the appendix (Table 6). Participants majorly reported that COVID cases are rising as people are not following the government rules properly, there is ignorance and lack of education among masses, people are taking it lightly by not wearing masks properly and not following social distancing appropriately, dense population in our country, non-disclosure of an infected person due to fear of being taken by police and ambulance, lack of facilities at government hospitals, overcharges and non-acceptance by private hospitals, lack of faith in government machinery, too much fear and anxiety being created by news channels rather than creating awareness among the general population.

The solutions suggested by participants to curb the spread of coronavirus include wearing masks properly, maintaining social distance, avoiding maximum gatherings at religious places, crowded places, malls, theatres, etc., adopting personal hygiene, frequent hand washing, staying at home and breaking the chain, consulting the doctor if any symptoms appear. The government should have facilities available for proper quarantine for immediate contacts, strict action must be taken against offenders and vaccinations are required as soon as possible.

Assessment of association between various characteristics and preventive practices

T-tests and one-way ANOVA/Kruskal-Wallis test were used to assess the differences in scores based on the response chosen for 18 questions among various socio-demographic characteristics shown in Table 4. The scores were significantly different across age groups (p-value = 0.008), gender (p-value < 0.05), marital status (p-value = 0.0024) and place of residence (p-value = 0.003). Higher scores were obtained among participants lying in the age group of 31-50 years (79.71 ± 7.80), female participants (80.49 ± 0.36), people who were married (79.49 ± 8.20), and people residing in metropolitan (79.32 ± 7.93) and small cities (79.12 ± 8.87). However, no significant difference (p-value = 0.64) was found among participants of different socioeconomic status.

**Table 4:** Association between various characteristics and preventive practices One-way ANOVA applied; *Kruskal-Wallis test applied; # t-test applied

Groups	Responses	Association
Age (years)		
18-30 (1)	77.35 ± 9.25	F (2,961) = 7.19, p-value = 0.008 p (1vs2) = 0.001 p (1vs3) = 0.027
31-50 (2)	79.71 ± 7.80	
50 and above (3)	79.30 ± 8.79	
Gender#		
Male	77.67 ± 0.39	t = -5.1062, p-value < 0.05
Female	80.49 ± 0.36	
Marital status*		
Single (1)	77.43 ± 9.25	χ^2 ^(2) = 12.024, p = 0.0024 p (1vs2) = 0.011
Married (2)	79.49 ± 8.20	
Others (3)	79.40 ± 10.16	
Socio-economic Status		
Upper	78.83 ± 8.47	F (2,961) = 0.44, p-value = 0.64
Middle	79.18 ± 8.04	
Lower	78.55 ± 9.25	
Place of Residence		
Metropolitan cities (1)	79.32 ± 7.93	F (2,961) = 5.89, p-value = 0.003 p (1vs3) = 0.005
Small cities (2)	79.12 ± 8.87	
Small towns and villages (3)	76.53 ± 9.93	

## Discussion

COVID-19 seems to have an impact on various aspects, whether it is psychological [10], social or behavioural [11,12]. Considering the fact that the pandemic might still continue in future and stringent adoption of preventive practices is the only crucial way to curb the spread of the disease, assessment of the extent of adoption of preventive practices against COVID-19 among citizens is of paramount importance. Timely and regular identification of the reasons for the lapse in following these practices religiously might be helpful in formulating effective strategy and policy.

We used a scientifically developed and validated questionnaire to evaluate the preventive practices followed by the general population against COVID-19. It was found that people were wearing masks when going out but a considerable proportion of them were not stringent in wearing it properly and while doing so they missed out covering their mouth and nose mainly due to difficulty in breathing or the mask slides down due to its loose fit. Moreover, many were not aware of how to exactly dispose of these used masks. It is required that masks should be made available to the public that are of appropriate fit and increased awareness should be spread among people regarding how to deal with the used masks. People also avoided shaking hands in the current scenario but when it came to maintaining social distance lesser number of people were doing that as they feel that there is lack of space. Similarly, a lesser number of people reported frequent hand washing for adequate duration as guided by health authorities which might be because of ignorance and lack of awareness. There is a need to make people more aware regarding the importance of hand washing and its appropriate duration.

Studies conducted across the world have assessed the knowledge, attitude and practices of the general population, not covering the preventive practices comprehensively [13-18]. Moreover, in India, studies assessing the adoption of preventive practices have targeted either specific subgroups or health care workers [15, 19]. Semi-structured questionnaires that have not been validated were used in these studies. We have used a well developed and validated questionnaire to evaluate the preventive practices against COVID-19. Certain findings of our study are in concurrence with other studies reporting that people were wearing masks when going out [19-21]. However, in contrast to these studies, our study reported a lesser number of people maintaining social distance at their workplace and in public places. This difference might be due to the fact that other studies were conducted during the earlier phase of COVID-19 whereas our study has been conducted recently during the unlock phase of our country but still officials need to make sure that social distancing is followed religiously, else heavy fines should be imposed on the ones violating the law. Our study also reported a lesser number of people washing their hands at regular intervals. This might be because people are now finding it cumbersome to wash hands so many times. In contrast to the study conducted by Azlan et al. where practices followed by people of low socio-economic group secured lower scores [21], in our sample we did not find any significant difference among the scores obtained by people of different socioeconomic groups. On the other hand, we found that people residing in the metropolitan and small cities secured higher points than people residing in small towns and villages. This shows that still there is a need to make them aware about the preventive practices against COVID-19 and advantages of following them.

There are certain implications of our findings. Corona virus spread could be curbed by not just carrying masks but also wearing it properly by covering the nose and mouth as advised by Centers for Disease Control and Prevention (CDC) [22]. Along with the proper use of masks it is crucial to wash hands thoroughly to prevent the cross-transmission of this virus [23]. Social distancing norm is yet another dimension that needs attention in the current pandemic times [5]. Though there has been a lot of coverage about these preventive practices in news, radio, advertisements and many public figures are promoting these practices but a lag is now being observed among people as they are getting tired following these practices and showing a carefree attitude especially during this unlock phase. This issue needs to be strictly addressed as it might lead to a greater spike in COVID cases in the coming period.

There are several strengths of our study. Firstly, we have used a well developed, validated and reliable tool to assess preventive practices being followed against COVID-19. In addition to this, our study is unique as it not only measures the preventive practices being followed but also explores the reasons for lapse in following these preventive practices, if any. Through this study we have been able to identify the weaker domains and the reasons for the same. The result of this study will help the health authorities and public health policy makers to identify various loops among the general population that are causing hindrance in curbing the spread of this disease. This will assist them in formulating other intervention strategies.

Our study has few limitations. Though we have covered participants from different zones of our country and socio-economic status, stratified sampling was not done. We have more participants from the urban areas as the participants were obtained through the snowball technique (contacts and networks of the investigators). Apart from this, as our study used a self-reported questionnaire, it might be possible that the participants answered the socially desirable responses.

## Conclusions

The study assisted in identifying attitude and behaviour of people across the country towards COVID-19 preventive practices during the post lockdown period. The study has raised the concerns that careless attitude in prevention of COVID can destroy the efforts of the nation. This study will prove to be useful for public health policy makers and health authorities to comprehend practices of the general public and evaluate if any stricter laws are required to be made. However, similar studies should be conducted at regular intervals to find out the loopholes in the preventive practices followed by the general public.

## Appendices

**Table 5:** Assessment of reasons for deficiency in following preventive practices

S No.	Question	Responses	Frequency of responses
Hand Hygiene			
	What is/are the reason(s) due to which you can’t avoid shaking hands in the current scenario (COVID-19 pandemic)?	Not applicable, Don't know that COVID spreads through hand shakes, Avoiding hand shaking will not prevent COVID infection, Difficult to change habit, Looks rude not do so when opposite person extends hand for handshake, Other reasons	751, 29, 20, 75, 113, -
	What is/are the reason(s) for not washing/sanitizing hands at frequent intervals?	Not applicable, Don't know that washing hands prevents spread of COVID, Frequent hand-washing will not prevent COVID infection, It leads to wastage of water and resources, Difficult to change habit, Non-availability/shortage of water/sanitizer, Lack of time, Cumbersome to sanitize hands too many times, Other reasons	668, 15, 12, 19, 92, 52, 74, 109, 10
	What is/are the reason(s) for not washing hands for at least 20 seconds?	Not applicable, Don’t know that hands have to be washed for at least 20 seconds, Time duration is not important while washing hands, Unable to check the time while washing hands, Cumbersome when washing hands multiple times, Other reasons	567, 44, 53, 261, 101, 3
	What is/are the reason(s) for not coughing/sneezing into the handkerchief/bent elbow?	Not applicable, Don’t know that coughing into elbow stops spread of infection to others, It is not important in preventing spread of disease, Sometimes I forget, Difficult to change habit, Other reasons	728, 22, 13, 190, 85, -
	What is/are the reason(s) for touching eyes/nose/mouth without cleaning hands?	Not applicable, Don’t know that touching eyes/nose/mouth with unclean hands can cause spread of COVID, Not important in preventing COVID, Don’t remember, Difficult to change habit, Other reasons	575, 22, 16, 230, 170, 6
Social Distancing			
	What is/are the reason(s) for not maintaining social distance in the workplace?	Not applicable, Don’t know that at least 1-2-meter distance should be maintained, Social distancing is not important in prevention of COVID, Lack of space, Difficulty in talking, Overcrowding, Other reasons	665, 16, 8, 163, 74, 108, 14
	What is/are the reason(s) for not maintaining at least one-meter distance while having food with colleagues?	Not applicable, Don’t know that at least 1-2-meter distance should be maintained, Social distancing is not important in prevention of COVID, Lack of space, Difficulty in talking, Overcrowding, Other reasons	718, 20, 5, 145, 60, 65, 8
	What is/are the reason(s) for going out of house?	Not applicable, Work, Grocery shopping, Walking/exercising, Socializing, Visiting religious places, Entertainment (Club, visiting friends, etc.), Other reasons	226, 385, 476, 201, 45, 27, 23, -
	What is/are the reason(s) for not maintaining social distancing in public spaces?	Not applicable, Don’t know that at least 1-2-meter distance should be maintained, Social distancing is not important in prevention of COVID, Lack of space, Difficulty in talking, Overcrowding, Other reasons	605, 19, 3, 225, 47, 85, 1
Masks			
	What is/are the possible reason(s) for not wearing masks while going out of home?	Not applicable, I didn’t know wearing mask prevents spread of COVID, I believe masks are useless, Lack of availability, Not comfortable, Difficult to breath, Doesn’t look good, Other reasons	839, 9, 7, 10, 56, 89, 3, 3
	What is/are the reason(s) for not covering both nose and mouth while wearing masks?	Not applicable, Don’t know that both nose and mouth have to be covered, Don’t find it useful, Have difficulty breathing while wearing it, Don’t feel comfortable wearing it, It slides down due to loose fit, Other reasons	780, 7, - 95, 74, 62, 2
	What is/are the reason(s) for not keeping mask properly in separate bags/bins after using it?	Not applicable, Don’t know it should be kept properly in a separate bag/bin, Don’t know how to dispose of the mask, Don’t feel it is important to keep it properly, Too tired after work, Don’t find a suitable place to dispose the mask, Other reasons	707, 53, 71, 24, 50, 85, -
Gadgets/Fomites			
	What is/are the reason(s) for not cleaning personal items (e.g., purse/mobile phone, etc.) when you reach home?	Not applicable, Don't know that I should clean it after work, Believe it is not useful, Non-availability of sanitizers, Not needed as there is no contact with COVID positive patients, Too tired to do so, Using sanitizer on personal items like mobile will damage it, Other reasons	574, 50, 35, 37, 100, 91, 121, -
	What precaution(s) do you take while purchasing groceries from local stores/vendors?	Not applicable, Opting for home delivery, Shopping at a time when it is less busy, Wearing a face mask, Carrying hand sanitizer or wipes with you, Using mobile pay/debit cards/credit cards for making payments, Buying 1-2 weeks-worth of groceries at a time, Others reasons	204, 285, 367, 549, 388, 289, 407, -
Technology and Law			
	Have you installed the AarogyaSetu app in your phone?	Yes, No, Initially installed then deleted	554, 317, 93
	What is/are the reason(s) for not using the AarogyaSetu app?	Not applicable, Don’t know about it, Don’t find it useful, Don’t have space in phone for it, Don’t have a smartphone, It drains battery, Other reasons	583, 96, 158, 53, 47, 33, -
	What is/are the reason(s) for not obeying government restrictions?	Not applicable, Don’t know about government restrictions, They are not effective, Other reasons	896, 31, 37, -
Preparedness			
	What is/are the reason(s) for not being aware of any helpline/authority/hospital to contact in case you develop symptoms of disease?	Not applicable, Don’t know that there is any helpline/authority, Prefer to treat oneself at home if develop symptoms, Other reasons	788, 39, 137, -

**Table 6:** Classification of responses obtained from participants to what do they think is the cause of the rising number of COVID cases among general population and their solutions

Causes for rising number of COVID cases as per participants responses		
S No.	Cause	Frequency of responses
1	Irresponsible behaviour of people. They have a careless attitude.	787
2	People are not sincerely following the rules of the government.	793
3	People are either not wearing masks or they are wearing it inappropriately.	689
4	People are not maintaining social distance.	605
5	People are not following hand hygiene properly.	430
6	People do not believe that the coronavirus does exist and are having faith on fake messages.	3
7	People want to rush back to work.	108
Solutions to control the rising number of cases		
S No.	Solutions	Frequency of responses
1	Wearing masks properly	602
2	Maintaining social distance	584
3	Avoiding maximum gatherings at religious places, crowded places, malls, theatres, etc.	346
4	Adopting personal hygiene, frequent hand washing	423
5	The government should have proper quarantine facilities.	183
6	Strict action must be taken against offenders.	554
7	Vaccinations are required.	276
